# Desire, Familiarity, and Engagement in Polyamory: Results From a National Sample of Single Adults in the United States

**DOI:** 10.3389/fpsyg.2021.619640

**Published:** 2021-03-23

**Authors:** Amy C. Moors, Amanda N. Gesselman, Justin R. Garcia

**Affiliations:** ^1^Department of Psychology, Chapman University, Orange, CA, United States; ^2^Kinsey Institute, Indiana University Bloomington, Bloomington, IN, United States

**Keywords:** consensual non-monogamies, consensual non-monogamous relationships, polyamory, polyamorous relationships, sexuality, family, romantic relationships

## Abstract

Coupledom and notions of intimacy and family formation with one committed partner are hallmarks of family and relationship science. Recent national surveys in the United States and Canada have found that consensually non-monogamous relationships are common, though prevalence of specific types of consensual non-monogamy are unknown. The present research draws on a United States Census based quota sample of single adults (*N* = 3,438) to estimate the prevalence of desire for, familiarity with, and engagement in polyamory—a distinct type of consensually non-monogamous relationship where people typically engage in romantic love and sexual intimacy with multiple partners. Results show that 1 out of 6 people (16.8%) desire to engage in polyamory, and 1 out of 9 people (10.7%) have engaged in polyamory at some point during their life. Approximately 1 out of 15 people (6.5%) reported that they knew someone who has been or is currently engaged in polyamory. Among participants who were not personally interested in polyamory, 1 out of 7 (14.2%) indicated that they respect people who engage in polyamory. Few sociodemographic correlates emerged; no differences in prevalence were found based on political affiliation, income, religion, geographic region, or race/ethnicity. Sexual minorities, men, and younger adults reported greater desire to engage in polyamory (compared to heterosexuals, women, and older adults, respectively). Men and people with lower education backgrounds were more likely to have previously engaged in polyamory (compared to women and people with higher education backgrounds, respectively). Given that emotional and sexual intimacy is an important part of most people’s lives, understanding the varied ways in which people navigate their intimate lives is critical for the fields of relationship, sexuality, and family science.

## Introduction

Coupledom and notions of intimacy and family formation with one committed partner are hallmarks of family and relationship science. Investigations of diverse intimate relationships—long-term cohabitation, blended families, and even affairs—abound within family and relationship science literature (e.g., Bumpass et al., 1991; Treas and Giesen, 2000; Schmitt, 2005; Carr and Springer, 2010). However, these inquiries almost exclusively focus on monogamous relationships, including predictors of longevity or divorce and instances of serial monogamy. In Western science, we tend to conceptualize romantic love as limited to only one person (a zero-sum allotment of love), yet we appear to view familial and platonic love as endless (Burleigh et al., 2017; Moors et al., 2019). Although there are a variety of ways in which people navigate their intimate lives, theories of human development and intimacy often implicitly assume that a preference for monogamy is universal (Seltzer, 2000; Conley et al., 2017; Moors et al., 2017). This rose-tinted scientific lens for monogamy raises the questions: How common is desire for and engagement in polyamory—a relationship structure in which partners engage in multiple loving and sexual relationships? How common is it to personally know someone who is/has engaged in polyamory? Do people respect polyamory as a relationship option?

While many people around the globe engage in serial monogamy, remarkable transformations in relationship and family demography have occurred over the past several decades (Glick, 1988; Fisher, 1989; Finkel et al., 2014; Foster, 2016). At the nexus of this change is the new reality of contemporary intimacy that has received limited attention by researchers, clinicians, and policymakers: A sizable portion of adults in the United States and Canada have been or are currently involved in consensually non-monogamous relationships (e.g., swinging, open, and polyamorous relationships; Haupert et al., 2017a; Fairbrother et al., 2019). Approximately 1 out of 22 people (4–5%) who are currently in a romantic relationship identify as part of a consensually non-monogamous relationship (4–5%; Fairbrother et al., 2019; Levine et al., 2018; Rubin et al., 2014). When asked about an ideal relationship type, approximately 1 out of 9 people (11.9%) indicate consensual non-monogamy (Fairbrother et al., 2019). Looking at lifetime prevalence, 1 out of 5 people (19.6–21.9%) have engaged in some form of consensual non-monogamy (Haupert et al., 2017a, b; Fairbrother et al., 2019). To put this into perspective, previous engagement in consensual non-monogamy is as common as owning a cat or speaking a language other than English at home in the United States (Newport et al., 2006; DeNavas-Walt et al., 2017).

Within the past couple of years, scholars have begun to shed light on national-level interest and engagement in consensual non-monogamy. What remains unknown is desire for and engagement in *specific* types of consensually non-monogamous relationships, such as polyamory. One study suggests that the general public’s interest in polyamory appears to be on the rise. Through an analysis of hundreds of thousands of people’s Internet searches, Moors (2017) found that seeking out information about polyamory has markedly increased over the past 10 years in the United States. Coinciding with the general public’s interest, media representation of polyamory has emerged over the past several years—from TV and film representation (e.g., Showtime’s Polyamory: Married and Dating; Netflix’s Insecure; Professor Marston and the Wonder Women) to mainstream news coverage (e.g., *New York Times*, *BBC*, *Buzzfeed*). However, understanding prevalence for desire, previous engagement, and familiarity remains unknown.

The goal of the present study is to establish prevalence estimates and understand sociodemographic correlates for (1) lifetime engagement in polyamory, including challenging and positive experiences, (2) willingness to engage in polyamory, (3) frequency of personally knowing someone who is/has engaged in polyamory, and (4) positive affect toward polyamory among people who have/would not personally engage in it. To our knowledge, this is the first study to focus on people’s desire for, engagement in, and familiarity with polyamory using a demographically representative sample of adults in the United States Examining prevalence of polyamory will advance our understanding of how Americans are transforming their intimate and family lives. In the next section, we provide an overview of typical relationship agreements for each of the common sub-types of consensual non-monogamy.

## Consensual Non-Monogamy: Boundaries and Definitions

Intimate relationships can be thought of as boundaries that people mutually agreed upon. Some people agree to be romantically exclusive (social monogamy) and sexually exclusive (sexual monogamy), whereas others may agree on varying levels of romantic and/or sexual openness (consensual non-monogamy; Conley et al., 2013; Gray and Garcia, 2013). At the core of consensually non-monogamous relationships are the consenting agreements to engage in varying degrees of romance and sex with more than one partner. That is, all partners involved make agreements to engage (or not) in concurrent romantic and/or sexual relationships (Conley et al., 2013; Moors et al., 2017). These relationships differ from infidelity or “cheating;” for this reason consensual non-monogamy is often referred to as ethical non-monogamy. Common forms of consensual non-monogamy include open relationships, swinging relationships, and polyamorous relationships. Swinging and open relationships tend to have boundaries that encourage sex with multiple concurrent partners but limit emotional intimacy or romantic love with these partners. Typically, people who engage in swinging partake in sexual activity with their partner (e.g., group sex, swapping partners; Buunk and van Driel, 1989; Matsick et al., 2014). People who engage in open relationships typically engage in sexual activity independently from their partner (Kurdek and Schmitt, 1986). Although, threesomes with one’s partner may be common among people in various types of consensually non-monogamous relationships, particularly for open relationships (Lehmiller, 2018; Scoats et al., 2018). While romantic love is typically “off-limits” for people engaged in swinging and open relationships, friendships appear to be common (Kimberly and Hans, 2017; Wood et al., 2018). Thus, it would be inappropriate to categorize these types of consensually non-monogamous relationships as solely “no strings attached” sexual relationships.

Distinct from swinging and open relationships, agreements in polyamorous relationships typically encourage romantic love and sexual activity with multiple concurrent partners (Barker, 2005; Moors et al., 2017). In the context of polyamory, romantic love and emotional closeness is often viewed as endless rather than limited to only one person (Moors et al., 2019). Polyamorous relationships are structured in a variety of ways, including one or two “primary” partners (often the focal or longest relationship partner) and additional “secondary” partner(s) (often referred to as hierarchical polyamory; Barker, 2005; Sheff and Tesene, 2015; Balzarini et al., 2019a). Polyamorous relationships may also take the form of triads (three person relationships), quads (four person relationships), or “V”s (Munson and Stelboum, 1999; Barker, 2005). Moreover, some polyamorous relationships are not open for everyone, *per se*, as “polyfidelity” refers to remaining sexually and romantically exclusive to a specific multi-person relationship. In addition, some people practice “mono-poly” relationships where one partner identifies as monogamous and the other partner has romantic/sexual relationships with multiple people (Sheff, 2016). Although the prevalence of people who identify as asexual and engage in polyamory is unknown, romantic/emotional intimacy without sexual intimacy can also exist within polyamory (Klesse, 2006; Scherrer, 2010).

## Interest and Engagement in Consensual Non-Monogamy

As mentioned above, previous engagement in consensual non-monogamy among people in the United States and Canada is common (Haupert et al., 2017a; Fairbrother et al., 2019). Moreover, consensual non-monogamy is practiced by a wide range of people. In a study of two large demographically representative samples of single United States adults (*N* = 3,905 and *N* = 4,813), Haupert et al. (2017a) found few sociodemographic predictors of lifetime engagement in consensual non-monogamy. Specifically, past engagement in consensual non-monogamy did not vary significantly by age, education level, income, religion, political affiliation, geographic region, or race/ethnicity. Only two sociodemographic differences emerged. Men, compared to women, were more likely to have previously engaged in consensual non-monogamy (OR 1.66–1.83 times more likely). Moreover, lesbian, gay, and bisexual individuals had previously engaged in consensual non-monogamy at a higher frequency than heterosexual individuals (OR 1.59–2.02 times more likely). This pattern of results for gender as well as non-significant findings for age, income, and race/ethnicity were also found in a nationally representative sample of Canadian adults (other sociodemographic factors were not assessed; Fairbrother et al., 2019).

To provide a more nuanced understanding of previous engagement in polyamory, we also asked people about their experiences in these relationships, including reasons why people may have found polyamory challenging or if they would engage in polyamory again in the future. Qualitative research has documented two commonly mentioned challenges among people engaged in consensually non-monogamous relationships: managing jealousy and navigating multiple emotional bonds (Ritchie and Barker, 2006; Aguilar, 2013; Sheff, 2015; Rubinsky, 2018). For instance, people engaged in polyamory mention that they experience jealousy about their partner’s partners, however, they often describe jealousy in mild terms (developed new words such as “shaky” to describe this feeling; Ritchie and Barker, 2006). Similarly, some people describe difficulty having to unlearn traditional dating scripts (e.g., exclusivity, possessiveness) and, instead, engage in transparent and honest emotional relationships (Aguilar, 2013). Thus, in the present study, we investigated whether people experienced possessiveness and difficulty with emotions in the context of polyamory. To our knowledge, the present study is the first to capture whether people who have engaged in polyamory (or any type of consensually non-monogamous relationship) would do so again in the future.

While research on lifetime prevalence of consensual non-monogamy has provided much needed insight into diverse expressions of intimacy, research has yet to examine future intentions. In addition to understanding lifetime prevalence of polyamory in the present study, we probed whether people desire to engage in polyamory. Recent research using convenience samples has examined willingness or desire to engage in consensual non-monogamy (e.g., Moors et al., 2015; Cardoso et al., 2020). Moors et al. (2015) found that among heterosexually identified people who had never engaged in consensual non-monogamy, men expressed greater willingness than women to engage in all three popular consensual non-monogamy sub-types. In this study, people rated their willingness to engage in various consensual non-monogamous arrangements (e.g., may have sex and romantic relationships with whomever, but there must be no secrets) on a 7-point Likert scale. Looking at people who reported willingness ratings of 4 and above (somewhat to extremely), up to 31% of men and 16% of women expressed moderate-to-high levels of willingness (Moors et al., 2015). In another study, Moors et al. (2014) found that desire to engage in consensual non-monogamy was high among sexual minorities. Specifically, differences did not emerge based among bisexual men and women or gay/lesbian men and women. Looking at willingness to engage in different sexual practices, a nationally representative study of United States adults found sizable proportions of people (ranging from 11.6 to 22.1%) indicated that engaging in group sex, threesomes, and swingers parties were somewhat or very appealing (Herbenick et al., 2017). Moreover, men were more likely than women to express greater interest to engage in these three sexual practices. Although interest in sexual practices is not a direct assessment of willingness, these estimates help provide a proxy to interest in some types of consensual non-monogamy (e.g., swinging). Indeed, recent research has documented that positive attitudes toward consensually non-monogamous relationships predict greater willingness to engage in these relationships (Cardoso et al., 2020).

## Positive Affect Toward Consensual Non-Monogamy and Personal Networks

In the present study, we also examined whether people knew someone who is or has been in a polyamorous relationship as well as positive attitudes toward polyamory. Stigma toward people engaged in consensually non-monogamous relationships is well documented. Compared to monogamous relationships, consensually non-monogamous relationships are perceived as low in relationship quality, less committed, immoral, and harmful to children (Moors et al., 2013; Grunt-Mejer and Campbell, 2016; Rodrigues et al., 2018). Less understood, however, is if people who are not personally interested in polyamory hold positive attitudes toward polyamory. This type of positive affect could be considered a proxy to being an ally. In the present study, we assessed whether people who were not personally interested in polyamory respected this type of relationship option. In addition, we also examined whether people knew someone in their personal network who had or is currently engaged in a polyamorous relationship. To our knowledge the present study is the first to assess prevalence of personally knowing someone who practices polyamory.

## Present Study

The purpose of the present study was to establish prevalence estimates and investigate preferences for polyamory based on a United States Census-based national quota sample of single adults (unmarried or not in a current long-term relationship). While many married Americans may have engaged in polyamory, our focus on single adults allows for widely applicable results, as most United States adults are single for a substantial duration of time (U.S. Census Bureau, 2020). We sought to investigate how sociodemographic factors (e.g., gender, age, religion) were related to each study aim: (1) to establish lifetime prevalence of polyamorous relationships, including challenging and positive experiences related to polyamory and desire to engage in polyamory again in the future, (2) to investigate willingness to engage in polyamory, (3) to identify the frequency of personally knowing someone who is/has engaged in polyamory, and (4) to understand positive affect toward polyamory among people who have/would not personally engage in it.

Given that approximately one-fifth of adults in the United States have engaged in consensual non-monogamy during their lifetime (Haupert et al., 2017a), we anticipate that engagement in polyamory, a sub-type of consensual non-monogamy, will be also be relatively common (though, a smaller proportion than one-fifth). We also anticipate that estimates of willingness to engage in polyamory will be greater than lifetime prevalence. Additionally, as previous research has consistently shown that gender and sexual orientation are related to previous and current engagement in consensually non-monogamous relationships (Haupert et al., 2017a, b; Levine et al., 2018; Fairbrother et al., 2019), we expect that men (compared to women) and sexual minorities (compared to heterosexual individuals) will have engaged in polyamory at some point during their life at a higher frequency, and will express greater interest to engage in polyamory.

Further, we explore the frequency of personally knowing someone who has practiced polyamory, as well as various challenges (e.g., possessiveness) and positive experiences (e.g., future relationships will only be polyamorous) reported by people who have engaged in polyamory. Because these are both exploratory investigations, we do not have specific predictions.

Last, we do not have theoretical reasons to anticipate differences based on age, education level, household income, religious affiliation, race/ethnicity, or geographic region. Similar to previous United States and Canada national-level research on consensual non-monogamy (e.g., Haupert et al., 2017a; Fairbrother et al., 2019), we anticipate that few sociodemographic differences in lifetime prevalence and willingness to engage in polyamory will emerge.

## Method

### Data Collection

Data were collected as part of the annual *Singles in America* (SIA) study^1^. Inclusion criteria for the study included: being at least 18 years old, fluent in English, and single relationship status (i.e., unmarried and single, defined as not seeing anyone or dating casually). SIA is sponsored by the relationship company Match; however, participants were *not* recruited or in any way drawn from the Match population or subsidiary sites. Participants were recruited exclusively by ResearchNow (Dallas, TX, United States), using independent opt-in Internet research panels for population-based cross-sectional survey. Panelists were initially drawn from a diverse pool of established participants who have been continuously recruited over several years from variety of venues, including paper and electronic mailings, referrals, corporate partnerships, and internet recruitment. Participants were recruited from these opt-in research panels, with recruitment targeting based on demographic distributions (i.e., age, gender, ethnicity, region, income) reflected in the most recent Current Population Survey conducted by the United States Bureau of the Census. The current study also included augmented oversampling of certain demographic categories, specifically gay men and lesbian women.

Research panelists within the sample frame received a recruitment message inviting them to participate for financial remuneration. Remuneration was determined by ResearchNow; the average compensation standard per time spent on the survey was approximately $5.00 USD. Participants received slightly different compensation depending on how needed their particular demographic subgroup was for the sample, which was monitored and balanced in real-time. To screen for inclusion criteria and ensure data quality, research panelists were required to verify their identity through a certification process, which employs validation technologies in real-time to identify and screen out false, duplicate, unengaged, and unqualified respondents that may attempt to take a survey. Additionally, panelists were screened to ensure survey engagement, with those straight-lining responses or moving too quickly through panels removed. Participants completed the full survey, meaning there is no missing data. All data were collected over the Internet. Data access and analysis procedures were approved by [redacted for blind review] University’s Institutional Review Board.

### Questionnaires

The Singles in America survey included a wide variety of questions related to participants’ attitudes and behaviors around dating and relationships, in addition to information about demographic characteristics. For the purposes of the present study, the following subset of demographic items were examined: gender, age, sexual orientation, education level, household income, religious affiliation, political affiliation, race/ethnicity, and region. To assess desire, previous behavior, and attitudes toward polyamory, all participants were asked, “When it comes to polyamorous relationships (in a committed, sexual and romantic relationship with multiple people at the same time), which of the following are true for you?” Next, they were presented with the following 11 statements (see below). Participants responded by checking boxes next to all applicable statements. Checked boxes were coded as 1, unchecked as 0.

(1) I have been in a polyamorous relationship and I would be in another.
(2) I have been in a polyamorous relationship and I would not be in another.
(3) I have tried polyamory and found that I was too possessive to cope with it.
(4) I have been polyamorous but found that all of the emotional side effects were too difficult to navigate.
(5) I have never been in a polyamorous relationship but I would consider one.
(6) I have never been in a polyamorous relationship but I want to try it.
(7) I will only consider polyamorous relationships.
(8) I have fantasized about being in a polyamorous relationship.
(9) I would consider a polyamorous relationship if it was more socially acceptable.
(10) I know someone who has had/is in a polyamorous relationship.
(11) I respect polyamorous people but I could not do it myself.

To examine previous engagement in polyamory, we combined statements 1–4 to create an index of people who have tried polyamorous relationships (i.e., if they checked any of those 4 boxes, they were given a ‘1’ whereas if they did not check any, they were coded as ‘0’). Statements 5–9 were combined in the same way to create an index of people who indicated some desire to try or be in polyamorous relationships. Statement 10 was assessed individually to understand who has been exposed to polyamorous relationships in their personal social networks. Last, statement 11 was assessed individually to understand positive attitudes with people in polyamorous relationships, particularly for individuals who do not hold interest in personally engaging in these relationships. Note that 52 participants (0.9%) selected options that were contradictory at face-value (e.g., people who reported that they would not be in another polyamorous relationship, but also reported that they would consider only polyamorous relationships in the future). Because this was such a small subset of our sample, and because people’s feelings toward entering into polyamorous relationships in the future may be more complex than a yes or no, we chose to retain these individuals in the analyses.

### Participants

Extensive demographic information for participants (*N* = 3,438) is presented in Table 1.

**TABLE 1:** Sample demographics.

Variables	Mean or % of sample (*N* = 3,438)
Age	*M* = 42.93, *SD* = 17.42
**Gender**	
Men	42.1%
Women	57.9%
**Region**	
Midwestern United States	21.6%
Northeastern United States	18.3%
Southeastern United States	34.9%
Western United States	25.2%
**Ethnicity/race**	
Black/African American	17.6%
East Asian	4.5%
Hispanic/Latino	12.2%
Middle Eastern	0.4%
Native American/Alaskan Native	1.7%
South Asian	1.2%
White/Caucasian	67.2%
Other ethnicity	1.2%
**Sexual orientation**	
Bisexual	4.5%
Gay or lesbian	8.3%
Heterosexual	87.2%
**Religious affiliation**	
Agnostic	8.9%
Atheist	7.8%
Buddhist/Taoist	1.3%
Christian	62.1%
Hindu	0.3%
Jewish	4.1%
Muslim	0.7%
Spiritual but non-religious	9.5%
Other religion	5.3%
**Education level**	
High school diploma	10.7%
Vocational/technical degree	2.9%
Some college	21.9%
Associate’s degree	10.2%
Bachelor’s degree	35.0%
Graduate/professional degree	19.3%
**Household income**	
Less than $15,000	14.6%
$15,000–$29,999	21.4%
$30,000–$44,999	18.6%
$45,000–$59,999	16.2%
$60,000–$74,999	10.0%
$75,000–$99,999	9.9%
$100,000–$149,000	6.5%
$150,000 or more	2.8%
**Political viewpoint**	
Democrat	35.8%
Republican	64.2%

## Results

Below we describe our statistical approach to examine links between sociodemographic factors and each study aim: (1) previous engagement in polyamory, (2) willingness to engage in polyamory, (3) personally knowing someone who is/has engaged in polyamory, and (4) positive affect toward polyamory among people who have/would not personally engage in it. Next, we report national level frequencies of attitudes, desire, and behaviors related to polyamory followed by sociodemographic correlate analyses.

### Statistical Approach

To examine sociodemographic correlates of prior engagement in polyamory, willingness to engage in polyamory, knowing someone who has had or is currently in a polyamorous relationship, and positive affect toward polyamory among who are not personally interested in polyamory, we conducted four binary logistic regressions similar to the models conducted in Haupert et al. (2017a). Predictor variables included gender (coded as −0.5 = women, 0.5 = men), age (mean-centered), sexual orientation (coded as −0.67 = heterosexual, 0.33 = bisexual/gay/lesbian for LGB vs. heterosexual contrast; coded as 0 = heterosexual, −0.5 = gay/lesbian, 0.5 = bisexual for bisexual vs. gay/lesbian contrast), education level (mean-centered), household income (log-transformed for positive skewness), religious affiliation (5 codes, one for each affiliation; e.g., 0.5 = atheist, −0.5 = all others), political affiliation (Republican = −0.5, Democrat = 0.5), race/ethnicity (4 codes, one for each ethnicity; e.g., 0.5 = White, −0.5 = all others), and region (4 codes, one for each region; e.g., 0.5 = Midwest, −0.5 = all others). Note that because of cells equaling less than 20 participants, we did not include people who identified as South Asian or Middle Eastern, as Buddhist/Taoist, Hindu, or Muslim, or those who selected ‘other’ for their ethnicity or religious affiliation. Because we conducted a large number of comparisons on a large sample, we set the significant criterion for our tests to *p* ≤ 0.001 to protect against Type I error (see Cohen, 1992).

### Frequencies: Desire, Previous Engagement, Familiarity, and Positive Affect

Across the overall sample, 16.8% of participants reported desire to try or be in a polyamorous relationship, 10.7% reported previous engagement in polyamory, and 6.5% reported knowing someone who has been or is currently in a polyamorous relationship. Among participants who had previously engaged in polyamory, a sizeable portion (30.4%) would be in a polyamorous relationship again. Among participants who have previously engaged in polyamory, 21.1% indicated that they were too possessive to cope, and 32.8% indicated that the emotional aspects of polyamory were difficult to navigate. Among people who indicated that they are not personally interested in polyamory, 14.2% reported positive attitudes toward people in polyamorous relationships; see Table 2.

**TABLE 2:** Percentages of prevalence of polyamory: Previous engagement, desire, familiarity, and positive attitudes.

Construct	% of sample (*N* = 3,438)
**Previous engagement in polyamory**	10.7%
I have been in a polyamorous relationship and I would be in another	30.4%
I have been in a polyamorous relationship and I would not be in another	29.3%
I have tried polyamory and found that I was too possessive to cope with it	21.1%
I have been polyamorous but found that all of the emotional side effects were too difficult to navigate	32.8%
**Desire to engage in polyamory**	16.8%
I have never been in a polyamorous relationship but I would consider one	6.9%
I have never been in a polyamorous relationship but I want to try it	4.0%
I will only consider polyamorous relationships	2.4%
I have fantasized about being in a polyamorous relationship	4.9%
**Know someone who is/has engaged in polyamory**	6.5%
**Respect polyamory (but not personally interested)**	14.2%

*Percentages do not sum to total; participants could select multiple options. Indices were created if at least one option was selected.*

### Sociodemographic Correlates: Desire, Previous Engagement, Familiarity, and Positive Affect

**Previous engagement in polyamory.** All regression coefficients are presented in Table 3. Only gender and education level were significantly related to past engagement in polyamory at the *p* ≤ 0.001 level. Men were over twice as likely as women to report prior engagement, along with people with lower education levels (vs. higher levels).

**TABLE 3:** Correlates of previous engagement in polyamorous relationships.

Variable	OR	95% CI	*p*
Intercept	0.86	–	<0.001
Gender	2.16*	1.72–2.73	<0.001
Age	0.99	0.99–1.00	0.087
Sexual orientation: Gay/Lesbian/Bisexual vs. Heterosexual	0.99	0.70–1.38	0.935
Sexual orientation: Bisexual vs. Gay/Lesbian	0.79	0.42–1.48	0.461
Education level	0.88*	0.82–0.95	0.001
Household income	1.37	0.89–2.11	0.153
Religious affiliation: Agnostic	0.73	0.43–1.22	0.726
Religious affiliation: Atheist	0.95	0.57–1.59	0.857
Religious affiliation: Christian	0.70	0.48–1.02	0.062
Religious affiliation: Jewish	0.98	0.51–1.86	0.942
Religious affiliation: Spiritual	0.83	0.51–1.36	0.465
Political affiliation: Republican vs. Democrat	0.88	0.68–1.14	0.321
Race/ethnicity: White	0.61	0.39–0.95	0.027
Race/ethnicity: Black/African-American	1.01	0.62–1.64	0.978
Race/ethnicity: East Asian	1.12	0.62–2.02	0.714
Race/ethnicity: Hispanic/Latino	0.80	0.49–1.29	0.354
Geographical region: Northeastern United States^a^	1.13	0.81–1.57	0.464
Geographical region: Midwestern United States	0.78	0.55–1.10	0.155
Geographical region: Southern United States	0.96	0.71–1.29	0.764

^a^The response category “Western United States” was left out of analyses for redundancy. *p ≤ 0.001.

**Desire to engage in polyamory.** All regression coefficients are presented in Table 4. Gender, age, and sexual orientation (heterosexual vs. gay/lesbian/bisexual) were significantly related to desire to engage in polyamorous relationships. Men were nearly three times more likely to report desire than were women. Younger people were more likely than older people to report desire for engagement (although, this was a relatively small effect). In addition, people who identified as a sexual minority were over twice as likely as heterosexual participants to report desire to engage in polyamory.

**TABLE 4:** Correlates of desire to engage in polyamorous relationships.

Variable	OR	95% CI	*p*
Intercept	0.42	–	0.027
Gender	2.97*	2.43–3.63	<0.001
Age	0.98*	0.98–0.99	<0.001
Sexual orientation: Gay/Lesbian/Bisexual vs. Heterosexual	2.17*	1.69–2.78	<0.001
Sexual orientation: Bisexual vs. Gay/Lesbian	0.55	0.35–0.87	0.010
Education level	0.94	0.89–1.01	0.075
Household income	1.07	0.74–1.53	0.733
Religious affiliation: Agnostic	0.89	0.57–1.37	0.583
Religious affiliation: Atheist	1.58	1.03–2.42	0.034
Religious affiliation: Christian	0.69	0.49–0.96	0.028
Religious affiliation: Jewish	0.99	0.57–1.75	0.982
Religious affiliation: Spiritual	0.91	0.59–1.41	0.683
Political affiliation: Republican vs. Democrat	0.81	0.65–1.01	0.814
Race/ethnicity: White	0.86	0.60–1.25	0.433
Race/ethnicity: Black/African-American	1.36	0.91–2.04	0.135
Race/ethnicity: East Asian	0.93	0.54–1.59	0.781
Race/ethnicity: Hispanic/Latino	1.16	0.79–1.71	0.447
Geographical region: Northeastern United States^a^	1.10	0.82–1.47	0.512
Geographical region: Midwestern United States	1.12	0.84–1.49	0.427
Geographical region: Southern United States	1.11	0.86–1.43	0.435

^a^The response category “Western United States” was left out of analyses for redundancy. *p ≤ 0.001.

**Knowing someone who is/had engaged in polyamory.** All regression coefficients are reported in Table 5. Age and sexual orientation (heterosexual vs. gay/lesbian/bisexual) were significantly related to exposure to people engaging in polyamory in one’s personal social network. Younger participants were more likely to report knowing someone practicing polyamory (or has in the past), and those identifying as sexual minorities were nearly twice as likely as heterosexual participants to report knowing someone who had or is currently engaged in polyamory.

**TABLE 5:** Correlates of knowing someone who has tried polyamory.

Variable	OR	95% CI	*p*
Intercept	0.11	–	<0.001
Gender	1.42	1.06–1.91	0.020
Age	0.98*	0.97–0.99	<0.001
Sexual orientation: Gay/Lesbian/Bisexual vs. Heterosexual	1.87*	1.31–2.66	0.001
Sexual orientation: Bisexual vs. Gay/Lesbian	1.10	0.58–2.09	0.775
Education level	1.13	1.02–1.25	0.016
Household income	0.99	0.58–1.69	0.961
Religious affiliation: Agnostic	0.72	0.39–1.35	0.309
Religious affiliation: Atheist	1.30	0.73–2.33	0.375
Religious affiliation: Christian	0.58	0.36–0.94	0.026
Religious affiliation: Jewish	0.99	0.47–2.11	0.983
Religious affiliation: Spiritual	1.06	0.59–1.90	0.844
Political affiliation: Republican vs. Democrat	1.10	0.78–1.53	0.596
Race/ethnicity: White	1.19	0.70–2.00	0.521
Race/ethnicity: Black/African-American	1.30	0.72–2.32	0.382
Race/ethnicity: East Asian	1.05	0.49–2.25	0.910
Race/ethnicity: Hispanic/Latino	1.21	0.70–2.09	0.487
Geographical region: Northeastern United States^a^	0.97	0.64–1.49	0.900
Geographical region: Midwestern United States	1.08	0.72–1.63	0.716
Geographical region: Southern United States	0.97	0.66–1.41	0.853

^a^The response category “Western United States” was left out of analyses for redundancy. *p ≤ 0.001.

**Positive affect toward polyamory among people who were not personally interested in polyamory.** All regression coefficients are reported in Table 6. Age, sexual orientation (heterosexual vs. gay/lesbian/bisexual), and political affiliation were significantly related to positive affect toward polyamory among people who did not have personal interest in polyamory. Younger (vs. older) participants, sexual minorities (vs. heterosexuals), and Democrats (vs. Republicans) were more likely to report that they respect people who practice polyamory (even if they were not personally interested in polyamory).

**TABLE 6:** Correlates of “I respect polyamorous people but I couldn’t do it myself.”

Variable	OR	95% CI	*p*
Intercept	0.22	–	<0.001
Gender	0.91	0.73–1.12	0.370
Age	0.99*	0.98–0.99	<0.001
Sexual orientation: Gay/Lesbian/Bisexual vs. Heterosexual	1.68*	1.29–2.20	<0.001
Sexual orientation: Bisexual vs. Gay/Lesbian	1.23	0.75–2.02	0.417
Education level	1.10	1.03–1.18	0.008
Household income	0.78	0.53–1.13	0.189
Religious affiliation: Agnostic	1.40	0.91–2.17	0.130
Religious affiliation: Atheist	1.11	0.70–1.76	0.651
Religious affiliation: Christian	0.77	0.54–1.11	0.162
Religious affiliation: Jewish	0.89	0.49–1.59	0.687
Religious affiliation: Spiritual	0.92	0.59–1.44	0.714
Political affiliation: Republican vs. Democrat	1.61*	1.26–2.05	<0.001
Race/ethnicity: White	1.14	0.78–1.66	0.495
Race/ethnicity: Black/African-American	1.03	0.68–1.57	0.889
Race/ethnicity: East Asian	0.96	0.56–1.66	0.888
Race/ethnicity: Hispanic/Latino	1.18	0.79–1.75	0.419
Geographical region: Northeastern United States^a^	0.87	0.64–1.17	0.344
Geographical region: Midwestern United States	0.99	0.74–1.32	0.944
Geographical region: Southern United States	0.77	0.59–1.00	0.050

^a^The response category “Western United States” was left out of analyses for redundancy. *p ≤ 0.001.

## Discussion

Given the centrality of relationships and family, changes in these patterns have powerful implications for social life. Adding to a growing body of research on diverse expressions of intimacy and family life, we examined previous engagement in polyamory, willingness to engage in polyamory, personally knowing someone who engages in polyamory, and positive affect toward polyamory in a national sample of United States adults. We expanded previous research on the prevalence of consensual non-monogamy in several novel ways. Our results are the first to document prevalence estimates related to polyamory in particular. Specifically, we found that willingness to engage in polyamory and previous engagement in polyamory is common. Approximately 1 out 6 people desire to engage in polyamory and 1 out of 9 people have engaged in polyamory at some point during their life. To help put this into perspective, desire to engage in polyamory is as common as how many Americans would like to move to another country (Espipova et al., 2018), and previous engagement in polyamory is as common as holding a graduate degree in the United States (United States Census Bureau, 2019). Moreover, approximately 1 out of 15 people know someone in their social network who is currently or has in the past engaged in polyamory. Among people in the present study who were not personally interested in polyamory, 14.2% of people reported that they respect people who engaged in polyamory. That is, the majority of people who were not personally interested in polyamory did not indicate positive attitudes toward polyamory.

We also found that desire to engage and previous engagement in polyamory is common among people from a range of diverse racial, political, income, religious, and geographic backgrounds. In fact, we found few links between sociodemographic factors and desire or previous engagement in polyamory. Of the few differences documented, people who identified as lesbian, gay, or bisexual (compared to people who identified as heterosexual) and men (compared to women) were more likely to report desire to engage in polyamory and previous engagement in polyamory (consistent with our hypotheses). Lesbian, gay, and bisexual individuals may be more inclined to desire polyamory because questioning a heteronormative model of relationships encourages considering alternative relationships styles (Klesse, 2016). Moreover, given engagement in consensual non-monogamy is higher among lesbian, gay, and bisexual people (compared to heterosexuals; Haupert et al., 2017a, b), having familiarity with or learning norms about consensual non-monogamy may reduce stigma toward these types of relationships among people. In terms of men’s, relative to women’s, high willingness to engage in polyamory, some scholars suggest that this reported desire is an artifact of gendered dating norms (Moors et al., 2015) while others suggests this finding illustrates evolutionary mechanisms for human mating (Mogilski et al., 2017). We also found that younger people, compared to older people, were more likely to indicate willingness to engage in polyamory (inconsistent with our predictions). Desire to try polyamory among younger adults could be related to younger adults’ tendency to hold progressive values (e.g., sex positive views, diversity values; Regnerus and Uecker, 2011; Parker et al., 2019), and potentially to younger adults being the target audience for various media that have recently depicted polyamory.

In terms of previous engagement, we found that men were more likely than women to have previously engaged in polyamory at some point during their life (consistent with our hypotheses and previous research on consensual non-monogamy; Haupert et al., 2017a; Fairbrother et al., 2019). Inconsistent with our predictions, however, was that people who identify as a sexual minority or as heterosexual are equally likely to have previously engaged in polyamory. Although previous research indicates that sexual minorities are more likely (compared to heterosexuals) to engage in consensual non-monogamy (Haupert et al., 2017a), this was not found when looking at polyamory specifically. Perhaps among sexual minorities, higher levels of previous engagement in consensual non-monogamy may be related to engagement in open relationships (which could drive the difference based on sexual orientation when looking at all consensually non-monogamous relationships). Earlier research that used convenience sampling have documented that gay men, in particular, tend to use the term ‘open relationship’ and focus on sexual relationships with other partners (e.g., Blasband and Peplau, 1985; Kurdek and Schmitt, 1986). Inconsistent with our predictions, we found that people with lower education levels (high school and some college) were more likely than people with higher educational levels to have previously engaged in polyamory. This finding is also inconsistent with speculations from researchers that people with higher education levels may have had more exposure to information about polyamory or more financial stability to pursue multiple relationships (Sheff and Hammers, 2011). In the United States, approximately 33% of people have earned higher levels of education (a bachelor’s degree or higher; U.S. Census Bureau, 2019). Thus, most people in the United States, have completed some college or high school. The finding that lower education levels are associated with previous engagement in polyamory could reflect that the majority of people in the United States hold high school diplomas or some college experiences (as opposed to college and beyond experiences).

A common stereotype about consensual non-monogamy is that these relationships yield high jealousy and are challenging (Moors et al., 2013; Grunt-Mejer and Campbell, 2016). Indeed, qualitative research has documented that similar themes are expressed by people in consensually non-monogamous relationships, especially those new to them (e.g., Aguilar, 2013). In the present study, we found that between 21 and 33% of people who had previously engaged in polyamory experienced issues with their own possessiveness and difficulty with navigating their related emotions. Although these are sizable minorities, we have no way of knowing whether jealousy is more prevalent in polyamorous versus monogamous relationships, as there are no population-based studies of jealousy available. However, prior research using large convenience samples have documented that people engaged in monogamy report higher levels of jealousy than people engaged in consensually non-monogamous relationships (e.g., Conley et al., 2017). Moreover, research has shown that jealousy is a common experience in monogamous relationships. Jealousy is one of the leading predictors of divorce in longitudinal studies (Amato and Rogers, 1997), and using data from the General Social Survey, researchers found that between 32 and 46% of separated or divorced women reported that their ex-husbands were sexually jealous and/or possessive (Brownridge et al., 2008). Further, research conducted using twin studies has suggested that the propensity for romantic and sexual jealousy is somewhat heritable, indicating a person-level factor independent of any relationship arrangement (Walum et al., 2013). Although multi-partner relationship dynamics may provide more varied instances that could facilitate jealousy than would monogamous relationships, jealousy is likely present in all relationship types.

In terms of familiarity with polyamory, sexual minorities and younger adults were more likely to report that they knew someone who is/was engaged in a polyamorous relationship (compared to heterosexual individuals and older adults). Given that sexual minorities are more likely to have previously engaged in polyamory and other forms of consensual non-monogamy (e.g., Haupert et al., 2017a), it is not surprising that they are more likely than people who identify as heterosexual to know someone in their network who practices polyamory. Moreover, lesbian, gay, and bisexual individuals are less likely to adhere to rigid gendered norms surrounding dating, including desire for monogamy and marriage (Moors et al., 2014). There is also evidence that consensual non-monogamy is less stigmatized among lesbian, gay, bisexual, and queer people (Moors et al., 2013, 2014), and indeed, we found that sexual minority participants were more likely than heterosexual participants to indicate that they respect people engaged in polyamory. Specifically, these people who indicated that they were not personally interested in polyamory, but respect it as a relationship option. Future research could explore whether familiarity is linked with holding positive attitudes toward polyamory (akin to research on attitudes toward sexual minorities; Herek and Glunt, 1993), as well as with socio-demographics related to more socially liberal attitudes, as we found with younger participants and those who identified as Democrats. Another research direction could be to explore the extent to which people who are or have engaged in polyamory hold positive or negative views about polyamory. Recent research suggests that people engaged in consensual non-monogamy can hold self-stigmatizing views about their relationships style, similar to the psychological phenomena of internalized homophobia (Moors et al., in press).

In the next section, we provide a high-level overview of the growing area of scientific inquiry on consensually non-monogamous relationships. Beyond the scope of this paper is a critical review of the current literature. Instead, we provide context of some of the current research and how this body of work can be applied to relationship, sexuality, and family science. For further insight on theoretical and research implications of understanding consensually non-monogamous relationships, see reviews by Brewster et al. (2017), Conley et al. (2017), and Moors et al. (2017). For insight on inclusive research practices related to consensual non-monogamy, see Moors (2019).

### Future Directions and Implications for Relationship and Family Science

Finding a soulmate is central to mass media depictions of family life as well as social science theories of marriage and family. In fact, most people idealize monogamy and uphold a set of cultural assumptions that monogamous relationships are optimal and that monogamous romantic relationships should take priority over other relationships (known as mononormativity; DePaulo and Morris, 2005; Moors and Schechinger, 2014; Pieper and Bauer, 2014). That is, most people hold the belief that an exclusive coupled relationship is a “natural” part of the human experience and, subsequently, sexual behaviors outside of monogamous coupling are pathologized (a core concept related to queer theory; e.g., Rubin, 1984; Pieper and Bauer, 2014; De las Heras Gómez, 2019). The belief that monogamy is optimal is also an (implicit) assumption appears in many contemporary social science theories of intimacy, such as attachment theory and the investment model of relationships (e.g., Moors et al., 2015; Conley et al., 2017). One area ripe for future research is expanding relationship concepts and frameworks to include consensually non-monogamous relationship and family arrangements (see Olmstead, 2020, for a review focused on adolescence).

As found in the present study, societal views toward consensual non-monogamy tend to be negative and stigmatizing. Likewise, people engaged in consensual non-monogamy report a range of stigmatizing experiences based on their relationship (e.g., rejection from family and friends; child custody issues) and, often, hide their relationship style (Pallotta-Chiarolli, 2010; Sheff, 2015; Kimberly and Hans, 2017). These negative evaluations of consensual non-monogamy appear to be erroneous stereotypes. Research that has examined relationship qualities among people engaged in consensual non-monogamy and monogamy has generally found that people in both types of relationships report similar levels of relationship quality and psychological well-being (e.g., trust, commitment, love, depression; Rubel and Bogaert, 2015; Conley et al., 2017; Mogilski et al., 2017; Moors et al., 2017; Balzarini et al., 2019b). In some cases, people in consensually non-monogamous relationships report greater quality (e.g., lower jealousy, higher sexual satisfaction) and unique benefits, such as personal growth and diversified need fulfillment (Conley et al., 2017, 2018; Moors et al., 2017).

Furthermore, a growing body of research focused on relationship processes among people engaged in polyamory has documented a similar pattern of healthy relationship functioning. In terms of jealousy, people engaged in polyamory tend to experience low levels and use new words to describe mild forms of jealousy, such as “shaky” (Ritchie and Barker, 2006). Drawing on interpersonal relationship frameworks, Mitchell et al. (2014) investigated how meeting seven different needs (e.g., autonomy, closeness, emotional support, security) with a given partner affects relationship satisfaction and commitment with both relationship partners among people engaged in polyamory. Overall, need fulfillment across all needs were consistently high with both partners; moreover, the extent to which one partner met someone’s needs was unrelated to satisfaction or commitment with another partner. A similar pattern of results was found when looking at attachment dynamics and relationship quality among people engaged in polyamory (Moors et al., 2019). Specifically, Moors et al. found that people engaged in polyamory exhibited high levels of security with both of their partners (levels higher than established norms). Moreover, there was no association between avoidance and anxiety with one specific partner and the relationship functioning (e.g., satisfaction, commitment) in a different, concurrent relationship. These studies suggest that a relationship with one partner tends to function independently of a relationship with another partner, as both relationships were considered fulfilling, satisfying, and secure (essentially without influencing each other). In the context of the present studies’ findings, a future avenue to explore is the association between attachment bonds and reasons why some people thrive in polyamorous relationships while others experience jealousy or difficulty with navigating their emotions.

In the context of parenting, longitudinal sociological research illustrates the varied ways in which children raised by parents engaged in polyamory thrive (Sheff, 2011, 2015). For instance, children of parents engaged in polyamory report that they enjoy receiving attention from a variety of adults and sharing a diverse range of interests with adults in their lives (Sheff, 2010, 2015). In addition to benefits mentioned by children, parents engaged in polyamory expressed that multiple co-parents (or partners) helped with childrearing and household responsibilities. Although drawbacks such as breakups (and children reported that they missed these adults) can occur in polyamorous family units, this can be likened to feelings of loss that children of monogamous children experience when faced with divorce and separations. One limitation of the present study is that we did not examine whether people were parents and their experiences with or interest in polyamory. Future research could explore the extent to which people who are parents desire to or are engaged in polyamory.

To our knowledge, this study is the first to obtain information about the prevalence of polyamory, including previous engagement, desire, and familiarity, using a large United States national sample. Our study sheds light on the commonness of interest and previous engagement in polyamory among Americans. At the same time, our study focused on the experiences of people who are currently single, which limits the generalizability of our findings to people who are in relationships (including obtaining an estimate of current engagement in polyamory). Future research will benefit from understanding current engagement in polyamory as well as other specific types of consensual non-monogamy. Future research could also explore potential changes in desire or engagement in consensually non-monogamous relationships (or polyamory specifically) over time. A limitation of the present study is that it captures attitudes and behaviors related to polyamory at one time point.

## Data Availability Statement

The raw data supporting the conclusions of this article will be made available by the authors, without undue reservation.

## Ethics Statement

The studies involving human participants were reviewed and approved by Institutional Review Board, Indiana University. The patients/participants provided their written informed consent to participate in this study.

## Author Contributions

All authors contributed to writing and editing the manuscript. AM led writing of introduction and discussion. AG led data analysis. JG and AG designed the survey and collected the data.

## Conflict of Interest

The authors declare that this study received funding from the dating company, Match. Match was involved in the design of the full survey and funded the data collection, but did not collect the data. Match was not involved in the study design, analysis, interpretation of data, the writing of this article, or the decision to submit it for publication.

## Footnotes

1 A fully-detailed account of the methods for every wave of SIA can be found at www.kinseyinstitute.org/research/SIA.php
